# Synthesis, Characterization, and Biological Evaluation of Co(II), Cu(II), and Ni(II) Complexes With 2,2′‐Bipyridine‐5,5′‐Dicarboxamide Ligands: Antibacterial and Enzyme Inhibition Studies

**DOI:** 10.1002/open.70261

**Published:** 2026-07-15

**Authors:** İsmail Yılmaz, Müslüm Kuzu, Hasan Almohammed, Aseel Almadhoun, Hasan Solmaz, Raneem Mamoun Ali Nour

**Affiliations:** ^1^ Department of Chemistry Faculty of Engineering and Natural Sciences Karabük University Karabük Türkiye; ^2^ Department of Nutrition and Dietetics Faculty of Health Sciences Karabük University Karabük Türkiye; ^3^ Department of Nursing College of Nursing University of Telafer Nineveh Iraq; ^4^ Department of Medical Microbiology The Institute of Graduate Programs Karabük University Karabük Türkiye; ^5^ Department of Medical Microbiology Faculty of Medicine Karabük University Karabük Türkiye; ^6^ Department of Food Toxicology The Institute of Graduate Programs Karabük University Karabük Türkiye

**Keywords:** 2,2′‐bipyridine, amide ligands, antibacterial activities, enzyme inhibition, metal complexes

## Abstract

This study reports the synthesis and characterization of two novel 2,2′‐bipyridine‐5,5′‐dicarboxamide ligands (L1 and L2) and their corresponding Co(II), Cu(II), and Ni(II) complexes. The structures of the free ligands were verified using FTIR, NMR, UV–Vis, elemental analysis, and mass spectrometry, while the metal complexes were characterized through FTIR, MALDI‐TOF MS, UV–Vis, and magnetic susceptibility techniques. Single‐crystal X‐ray diffraction was employed to elucidate the structure of L1. Biological evaluation against resistant bacterial strains identified Co‐L2 as a potent antimicrobial agent, exhibiting a quantitative Minimum inhibitory concentration (MIC) of 500 μg/mL against *E. coli*, *P. aeruginosa*, and *S. aureus*. Furthermore, enzyme inhibition assays revealed that Ni‐L2 was the most effective inhibitor for pancreatic lipase (IC50 = 0.36 μM) and butyrylcholinesterase (IC50 = 4.53 μM). In contrast, the free L2 ligand demonstrated the strongest inhibitory potency against acetylcholinesterase (IC50 = 0.479 μM). These findings underscore the influence of 5,5′‐regiochemistry on the biological efficacy of bipyridine‐based systems.

## Graphical abstract

Synthesis of novel trinuclear [M_3_L_2_] complexes based on 5,5’‐regiochemistry is reported. Co‐L2 emerges as a potent lead with significant broad‐spectrum antibacterial activity. Ni‐L2 proves highly effective for pancreatic lipase and BChE inhibition, while the free L2 ligand exhibits superior AChE inhibitory potency. This work highlights how specific structural modifications modulate the biological efficacy of bipyridine‐based systems.

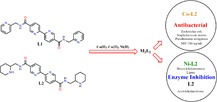

**Abbreviations**
bpy: 2,2′‐Bipyridine
CHN: Carbon, hydrogen, and nitrogen
ESI‐MS: Electrospray ionization mass spectrometry
FT‐IR: Fourier‐transform infrared spectroscopy
MALDI‐TOF: Matrix‐assisted laser desorption/ionization–time of flight
OAc: Acetate
py: Pyridine

## 1 Introduction

2,2′‐Bipyridine‐bodied ligands are among the most versatile building blocks in coordination chemistry, facilitating the design of metal complexes with a wide array of applications in material science, catalysis, and medicinal chemistry [1]. Transition metal complexes featuring pyridine derivatives have gained significant prominence due to their structural diversity and ability to enhance the stability and bioavailability of therapeutic molecules [2]. Recent developments emphasize the critical role of metal complexation in modulating biological activity, with studies reporting unique structural features and antibacterial potency in bipyridine‐based metal‐organic frameworks [3]. Furthermore, the synthesis of chiral complexes and mixed‐ligand systems derived from 2,2′‐bipyridine has demonstrated significant potential to improve antimicrobial efficacy against resistant pathogens [4].

While the coordination chemistry of these derivatives is well‐documented, current research focuses on modulating their pharmacological potential through precise structural modifications. In continuation of previous research on 2,2′‐bipyridine‐4,4′‐dicarboxamide ligands [5], the present study targets the 5,5′‐dicarboxamide regiochemistry. This specific substitution pattern is predicated on the expectation that the positioning of the amide groups at the 5,5′‐positions will significantly alter the electronic environment of the metal center and its subsequent interaction with biological targets, compared to the previously reported 4,4′‐analog. Numerous studies have demonstrated that such structural variations and the presence of amide functional groups are essential for exceptional biological activity and site‐specific recognition [6, 7].

The global escalation of antimicrobial resistance poses a critical threat to public health, necessitating the development of novel therapeutic agents that can disrupt intracellular metabolic processes. The enzymes that catalyze reactions essential to life are key targets for managing pathological disorders, as anomalies in specific enzyme activity occur in most metabolic conditions. For instance, pancreatic lipase inhibitors are vital for treating obesity by preventing fat absorption in the small intestine [8]. Recent investigations into pyridine‐containing metal complexes have highlighted their potential as effective lipase inhibitors through mechanisms such as active‐site hydrophobic complementarity [9].

The cholinergic system plays a central role in the pathogenesis of Alzheimer's disease (AD). According to the “cholinergic hypothesis,” acetylcholinesterase (AChE) and butyrylcholinesterase (BChE) are key therapeutic targets, as the rapid hydrolysis of acetylcholine leads to cognitive decline. Consequently, inhibiting these enzymes to halt disease progression has become one of the most significant therapeutic approaches in AD treatment [10, 11].

Previous studies have shown that the 4,4′‐bipyridine molecule inhibits AChE and BChE enzymes by interacting with them both catalytically and peripherally. The interaction between the molecule and AChE occurs via the tryptophan residues 86 and 286 [12]. Another study investigated the interactions of pyridine‐based Schiff bases with lipase, highlighting that residue interactions with p‐alkyl and hydrogen bonds in the active site could exhibit inhibitory effects [13]. These findings in the literature suggest that 2,2′‐bipyridine and its metal complexes may also affect these enzymes.

In this study, the synthesis, characterization, and biological evaluation of two novel 2,2′‐bipyridine‐5,5′‐dicarboxamide ligands and their corresponding Co(II), Cu(II), and Ni(II) complexes are reported. The objective is to elucidate how this specific substitution pattern influences their potential as antimicrobial agents and enzyme inhibitors.

## 2 Experimental Section

### 2.1 Materials and Instruments

All chemicals were purchased from commercial sources and used without further purification. 5,5′‐Dimethyl‐2,2′‐bipyridine and 2‐(aminomethyl)pyridine from Acros; nickel(II)‐acetate, 2‐(aminomethyl)piperidine, and benzene from Sigma‐Aldrich; NaOH from Carlo Erba; petroleum ether and K_2_Cr_2_O_7_ from Riedel‐de Haen; and copper(II) acetate monohydrate, thionyl chloride, H_2_SO_4_, dichloromethane, methanol, ethanol, and cobalt(II)‐acetate were purchased from Merck. The compounds were analyzed using Fourier‐transform infrared (FT‐IR) spectroscopy (Nicolet iS5, Thermo Scientific) using an attenuated total reflectance (ATR) accessory (iD7, Thermo Scientific). The elemental analyses of the ligands were performed using an LECO Truspec Micro CHN microanalysis apparatus with a LECO Truspec Micro. Melting points were measured with a Thermo Fisher Scientific IA9100 apparatus. The ^1^H‐NMR and ^13^C‐NMR spectra of the ligands were recorded on an Agilent 600 MHz spectrometer using DMSO‐*d*_6_ solvent. MS analysis of the ligands was performed using a Thermo Scientific TSQ Quantum Access MAX triple quadrupole mass spectrometer. Mass spectra of metal complexes were performed on a Bruker Microflex LT MALDI‐TOF MS. The electronic spectrum of the complex was measured using a UV‐Vis spectrophotometer (Genesys 10S UV–Vis, Thermo Scientific) in the range of 200–800 nm. Magnetic susceptibility measurements were performed on a Sherwood Scientific MX Gouy balance using Hg[Co(SCN)_4_] as calibrant. Crystals of L1 suitable for single‐crystal X‐ray diffraction were grown by slow evaporation of a methanol solution at room temperature. Details of data collection and crystal structure determinations are given in Table 1. Crystallographic data for the structural analysis of 1 have been deposited with the Cambridge Crystallographic Data Center, CCDC 2 320 725. The data were collected on a D8‐QUEST diffractometer equipped with graphite‐monochromatic Mo‐Kα radiation at 296 K. The structure was solved with OLEX2.solve [14] structure solution program using Charge Flipping and refined with the olex2.refine [14] using the Gauss‐Newton minimization package and SHELXL [15]. Molecular diagrams were generated using OLEX2 [16] and MERCURY [17].

**TABLE 1:** Crystal data and structure refinement for L1.

Empirical formula	C_24_H_24_N_6_O_4_
Formula weight	460.496
Temperature/K	296
Crystal system	Triclinic
Space group	P‐1
a/Å	7.4577(5)
b/Å	11.9258(7)
c/Å	13.4931(8)
α/°	70.639(2)
β/°	79.309(2)
γ/°	81.924(2)
Volume/Å^3^	1108.50(12)
Z	2
ρ_calc_ g/cm^3^	1.380
μ/mm^−1^	0.097
F(000)	484.3
Crystal size/mm^3^	0.31 × 0.3 × 0.16
Radiation	Mo Kα (λ = 0.71073)
2Θ range for data collection/°	3.64 to 56.6
Index ranges	−9 ≤ *h* ≤ 9, −15 ≤ *k* ≤ 15, −17 ≤ *l* ≤ 17
Reflections collected	31 069
Independent reflections	5489 [R_int_ = 0.0385, R_sigma_ = 0.0285]
Data/restraints/parameters	5489/0/403
Goodness‐of‐fit on F^2^	1.070
Final R indexes [I > = 2σ (I)]	R_1_ = 0.0441, wR_2_ = 0.1081
Final R indexes [all data]	R_1_ = 0.0612, wR_2_ = 0.1194
Largest diff. peak/hole/e Å^−3^	0.33/−0.22

### 2.2 Synthesis of the Compounds

#### 2.2.1 General Synthetic Procedure for 5,5′‐Dicarboxamide Ligands

The preparation of the ligands followed a multistep modified literature procedure [5, 18]. Initially, 5 g (27 mmol) of 5,5′‐dimethyl‐2,2′‐bipyridine was dissolved in 125 mL of concentrated H_2_SO_4_ at 70 °C. To this mixture, 24 g (82 mmol) of K_2_Cr_2_O_7_ was introduced dropwise until a dark green solution was obtained. The reaction was stirred for 5 h, after which it was diluted with 800 mL of ice‐water. The resulting yellow solid was collected, washed thoroughly, and subsequently refluxed in 150 mL of 50% HNO_3_ for 4h. Upon pouring back into ice‐water, the 2,2′‐bipyridine‐5,5′‐dicarboxylic acid was isolated as a white precipitate. For the amide coupling, 2 g (8.2 mmol) of the dicarboxylic acid was refluxed for 24 h in a mixture of 20 mL SOCl_2_ and 10 mL benzene. Following the removal of excess thionyl chloride and washing with petroleum ether, 1 g (3.6 mmol) of the resulting acid chloride in 40 mL dichloromethane was reacted with the appropriate amine (7.2 mmol) in 20 mL dichloromethane. The reaction was conducted at 0 °C with the addition of 20 mL of 0.5 M NaOH. The mixture was stirred for 5 h at 0 °C and then allowed to reach room temperature for an additional 19 h. The final white precipitates (L1 and L2) were filtered, washed with water, and purified via recrystallization from methanol.

#### 2.2.2 General Procedure for the Preparation of Metal Complexes

The metal complexes were synthesized by a direct complexation method at ambient temperature. Briefly, 0.1 g of the ligand (L1: 0.236 mmol or L2: 0.229 mmol) was dissolved in 30 mL of methanol. To this solution, an equimolar amount of the respective metal acetate salt namely copper(II) acetate monohydrate (0.047 g for L1; 0.046 g for L2), cobalt(II) acetate tetrahydrate (0.059 g for L1; 0.057 g for L2), or nickel(II) acetate tetrahydrate (0.059 g for L1; 0.057 g for L2) dissolved in 20 mL of methanol was added dropwise under stirring. The resulting colored solutions were stirred for 1 h to ensure complete coordination. The solvent was then removed under reduced pressure using a rotary evaporator. The residue was redissolved in 30 mL of deionized water, filtered to remove impurities, and slowly evaporated in open air to yield crystalline products. The final substances were collected and stored in a desiccator over anhydrous calcium chloride.

#### 2.2.3 5,5′‐Dicarboxy‐2,2′‐Bipyridine

FT‐IR spectrum [ATR/cm^−1^]: 3065, 2811, 2653, 2542, 1679, 1591, 1549, 1472, 1425, 1371, 1304, 1248, 1127, 1052, 1024, 935, 864, 816, 765, 690, 644. ^1^H NMR (400 MHz, DMSO) δ 13.45 (s, 1H, COOH); 9.14 (s, 1H, bpy6,6′); 8.50 (d, *J* = 8.3 Hz, 1H, bpy3,3′); 8.39 (d, *J* = 8.2 Hz, 1H, bpy4,4′). ESI‐MS (m/z): 244.90 (Calc. for C_12_H_8_N_2_O_4_ 244.21). Yield = 90%. M.p. 365 °C (decomposition).

#### 2.2.4 5,5′‐Bis(chlorocarbonyl)‐2,2′‐Bipyridine

FT‐IR spectrum [ATR/cm^−1^]: 3099, 3062, 1720, 1583, 1548, 1461, 1369, 1282, 1250, 1200, 1134, 1053, 1023, 870, 851, 737, 703, 662, 607, 521, 424. Yield = 91%. M.p. 322 °C.

#### 2.2.5 L1

FT‐IR spectrum [ATR/cm^−1^]: 3288, 3049, 2922, 1626, 1590, 1570, 1530, 1475, 1454, 1439, 1371, 1312, 1289, 1245, 1220, 1158, 1099, 1068, 1048, 1023, 996, 959, 937, 903, 851, 820, 764, 750, 731, 663, 642, 605, 545, 490, 420, 402. ^1^H NMR (600 MHz, DMSO‐*d*_6_) δ 9.41 (t, J = 5.9 Hz, 1H, NH), 9.19 (d, J = 2.5 Hz, 1H, bpy6,6′), 8.53 (d, J = 8.2 Hz, 1H, bpy3,3′), 8.51 (dd, J = 4.8, 1.8 Hz, 1H, py6,6′), 8.43 (dd, J = 8.2, 2.3 Hz, 1H, bpy4,4′), 7.76 (td, J = 7.7, 2.1 Hz, 1H, py4,4′), 7.37 (d, J = 7.8 Hz, 1H, py3,3′), 7.26 (dd, J = 7.6, 4.8 Hz, 1H, py5,5′), 4.61 (d, J = 6.0 Hz, 2H, CH_2_). ^13^C NMR and DEPT (151 MHz, DMSO) δ 165.09(C), 158.80(C), 156.86(C), 149.36(CH), 148.98(CH), 137.23(CH), 136.94(CH), 130.50(C), 122.64(CH), 121.56(CH), 121.08(CH), 45.20(CH_2_). Elemental analysis calcd for C_24_H_20_N_6_O_2_·H_2_O (%): C, 65.15; H, 5.01; N, 18.99; found: C, 66.01; H, 5.76; N, 19.03. ESI‐MS (m/z): 425.10 (Calc. for C_24_H_20_N_6_O_2_ 424.46). UV–Vis (MeOH, λmax nm): 204, 255, and 299. Yield = 68%. M.p. 213 °C.

#### 2.2.6 L2

FT‐IR spectrum [ATR/cm^−1^]: 3313, 3068, 2930, 2851, 1641, 1595, 1551, 1537, 1467, 1438, 1365, 1308, 1280, 1245, 1202, 1168, 1144, 1115, 1053, 1022, 981, 936, 907, 878, 854, 834, 812, 797, 759, 733, 720, 667, 643, 576, 549, 481, 463, 455. ^1^H NMR (600 MHz, dmso) δ 9.28–9.20 (m, 2H), 8.53–8.43 (m, 2H), 3.65–3.58 (m, 1H), 3.53 (dt, *J* = 14.1, 5.4 Hz, 1H), 3.41–3.18 (m, 2H), 2.83 (td, *J* = 12.9, 5.8 Hz, 1H), 1.82 (dq, *J* = 13.8, 3.6 Hz, 1H), 1.74 (dt, *J* = 13.1, 3.7 Hz, 1H), 1.71–1.60 (m, 2H), 1.54 (qd, *J* = 12.9, 3.6 Hz, 1H), 1.50–1.35 (m, 1H), 1.19 (dt, *J* = 27.0, 7.2 Hz, 1H). ^13^C NMR and DEPT (151 MHz, DMSO) δ 165.55(C), 156.76(C), 149.27(CH), 137.19(CH), 130.26(C), 120.83(CH), 55.94(CH), 44.34(CH_2_), 42.00(CH_2_), 26.29(CH_2_), 21.96(CH_2_), 21.86(CH_2_). Elemental analysis calcd for C_24_H_32_N_6_O_2_·5H_2_O (%): C, 54.74; H, 8.04; N, 15.96; found: 54.73; H, 7.43; N, 15.98. ESI‐MS (m/z): 437.00 (Calc. for C_24_H_32_N_6_O_2_ 436.56). UV–Vis (MeOH, λmax nm): 203, 250, and 299. Yield = 61%. M.p. 220 °C.

#### 2.2.7 Co‐L1 Complex [Co_3_(L1)_2_(H_2_O)_n_]⋅6OAc (1)

Color: Dark orange. FT‐IR spectrum [ATR/cm^−1^]: 3216, 3052, 2970, 1647, 1594, 1542, 1472, 1394, 1304, 1255, 1168, 1151, 1099, 1043, 996, 850, 751, 607, 524, 414. MALDI‐TOF MS (m/z): [L]^+^: 424.742; [L+Na]^+^: 446.540; [CoL(H_2_O)_2_(OAc)_2_]^+^: 636.827; [CoL_2_]^+^: 908.232; [Co_2_L_2_(H_2_O)_2_(OAc)_2_‐H]^+^: 1118.772; [Co_3_L_2_(H_2_O)_4_(OAc)_4_‐2H]^+^: 1330.592. UV–Vis (MeOH, λmax nm): 203, 256, and 303. M.p. >300 °C (decomposition).

#### 2.2.8 Cu‐L1 Complex [Cu_3_(L1)_2_(H_2_O)_n_]⋅6OAc (2)

Color: Dark green. FT‐IR spectrum [ATR/cm^−1^]: 3283, 3053, 2970, 1634, 1590, 1537, 1472, 1437, 1371, 1314, 1291, 1248, 1157, 1099, 1046, 1024, 996, 851, 820, 751, 652, 606, 544, 413. MALDI‐TOF MS (m/z): [L]^+^: 424.333; [L+Na]^+^: 446.385; [CuL‐H]^+^: 486.700; [CuL_2_]^+^: 912.734; [Cu_2_L_2_]^+^: 975.853; [Cu_3_L_2_‐H]^+^: 1038.266. UV–Vis (MeOH, λmax nm): 204, 260, and 313. M.p. >300 °C (decomposition).

#### 2.2.9 Ni‐L1 Complex [Ni_3_(L1)_2_(H_2_O)_n_]⋅6OAc (3)

Color: Green. FT‐IR spectrum [ATR/cm^−1^]: 3235, 3057, 2970, 1657, 1594, 1537, 1471, 1410, 1313, 1254, 1217, 1151, 1099, 1037, 1002, 857, 749, 658, 612, 524, 414, 408. MALDI‐TOF MS (m/z): [LH]^+^: 425.134; [NiL_2_+H]^+^: 908.041; [Ni_2_L_2_ + 2H]^+^: 968.762; [Ni_3_L_2_ + 2H]^+^: 1027.382. UV–Vis (MeOH, λmax nm): 204, 261, and 311. M.p. >300 °C (decomposition).

#### 2.2.10 Co‐L2 Complex [Co_3_(L2)_2_(H_2_O)_n_]⋅6OAc (4)

Color: Dark orange. FT‐IR spectrum [ATR/cm^−1^]: 3235, 3061, 2933, 2857, 1645, 1543, 1471, 1393, 1318, 1169, 1140, 1034, 855, 751, 647, 615, 539, 420, 408. MALDI‐TOF MS (m/z): [LH]^+^: 437.251; [CoL(H_2_O)_2_(OAc)_2_]^+^: 649.462; [Co_3_L_2_(H_2_O)(OAc)_6_ +0.5 H_2_O]^+^: 1430.465 (tentative artifact). UV–Vis (MeOH, λmax nm): 202, 253, and 302. M.p. >300 °C (decomposition).

#### 2.2.11 Cu‐L2 Complex [Cu_3_(L2)_2_(H_2_O)_n_]⋅6OAc (5)

Color: Green. FT‐IR spectrum [ATR/cm^−1^]: 3268, 3066, 2932, 2855, 1633, 1549, 1434, 1386, 1318, 1206, 1168, 1143, 1044, 854, 808, 752, 652, 616, 528, 474, 421, 402. MALDI‐TOF MS (m/z): [L]^+^: 436.820; [Cu_2_L(H_2_O)_2_(OAc)_4_+H]^+^: 837.020; [Cu_2_L_2_(H_2_O)_2_ +0.5 H_2_O]^+^: 1044.602 (tentative artifact) [Cu_3_L_2_)(H_2_O)_2_(OAc]^+^: 1159.568. UV–Vis (MeOH, λmax nm): 202, 254, and 311. M.p. >300 °C (decomposition).

#### 2.2.12 Ni‐L2 Complex [Ni_3_(L2)_2_(H_2_O)_n_]⋅6OAc (6)

Color: Light green. FT‐IR spectrum [ATR/cm^−1^]: 3242, 3052, 2932, 2857, 1645, 1605, 1546, 1474, 1391, 1335, 1205, 1169, 1140, 1083, 1036, 919, 855, 752, 647, 616, 535, 430. MALDI‐TOF MS (m/z): [LH]^+^: 437.667; [Ni_2_L]^+^: 554.151; [Ni_2_L(H_2_O)(OAc)_4_ + 0.5 H_2_O]^+^: 818.921(tentative artifact); [NiL_2_]^+^: 933.815; [Ni_2_L_2_‐H]^+^: 989.432; [Ni_3_L_2_)(H_2_O)_2_(OAc‐H]^+^: 1143.074. UV–Vis (MeOH, λmax nm): 202, 256, and 310. M.p. >300 °C (decomposition).

### 2.3 In Vitro Activities

#### 2.3.1 Bacterial Strain and Minimum Inhibitory Concentration

The antibacterial properties of Co‐L1, Cu‐L1, Ni‐L1, Co‐L2, Cu‐L2, and Ni‐L2 compounds were investigated in *Pseudomonas aeruginosa* (ATCC 27 853), *Escherichia coli* (ATCC 70 028), and *Staphylococcus aureus* (ATCC 29 213) strains. Methods detailed in a previous study were followed to isolate single colonies from stock cultures and determine the MIC for compounds exhibiting antibacterial activity [5]. The accurate bacterial concentration was determined spectrophotometrically by measuring the optical density (OD) at 600 nm. The target OD was set to 0.063. To determine the MIC, stock solutions of 2000, 1000, and 500 ppm (w/v) were prepared using sterile distilled water for substances exhibiting antibacterial activity. Measurements were taken using a 96‐well microplate. Readings were taken after the initial measurement at the second, fourth, and sixth hours, and at the end of the first and second days. All antibacterial assays were performed in triplicate to ensure reproducibility. Gentamicin [19] was employed as a standard reference antibiotic (positive drug control) to evaluate the relative potency of the synthesized compounds. The MIC value for Gentamicin was determined under identical experimental conditions for comparison with the metal complexes.

#### 2.3.2 Enzyme Inhibition Assay

For AChE and BChE activity measurements, a method described in a previous study was used with minor modifications [20]. Acetylthiocholine iodide was used as the substrate in these measurements. For lipase enzyme activity measurement, the method described by Bulut [21] was used. Measurements were taken at different concentrations of the synthesized ligands and complexes to determine their effects on enzyme activities. Activities measured in a medium without the synthesized molecules were taken as 100% (control measurement). From measurements taken in media containing different concentrations of the substances, % activity values were calculated relative to the control and plotted against each substance's concentration. Using this graph, IC_50_ values (inhibitory concentrations that reduce activity to 50%) were calculated for substances that showed an effect [22].

#### 2.3.3 Docking Studies

This investigation used the Schrödinger Molecular Modeling suite (Maestro 12.5) for the computational analyses. The workflow incorporated two principal computational modules: Emodel, which optimizes ligand conformation through energy evaluation, and GlideScore, which quantifies ligand‐binding affinity. The three‐dimensional crystallographic structures of the target proteins were sourced from the RCSB Protein Data Bank, with specific entries for pancreatic lipase (1LPB), AChE (4TVK), and BChE (4BDS). These structural datasets were imported into Maestro Release 2023‐1 for subsequent processing. Protein system preparation, essential for molecular docking simulations, was performed using the integrated Protein Preparation Wizard in the Schrödinger suite. The active site of the prepared enzyme was identified using the Receptor Grid Generation Application in Maestro. The chemical structures of the ligands were generated using ChemDraw 18.0. The molecular structures of the reference enzyme inhibitors were retrieved from the PubChem database (www.pubchem.ncbi.nlm.nih.gov) and subsequently imported into the modeling environment. Prior to docking, all compounds were prepared using the LigPrep module implemented in Maestro. Molecular docking calculations were then performed using the Glide XP protocol. For each ligand, the pose with the lowest binding free energy was selected for further analysis. Finally, interaction maps and binding mode visualizations were generated using Maestro Release 2023‐1.

## 3 Results and Discussion

### 3.1 Synthesis and Characterization

**SCHEME 1:**
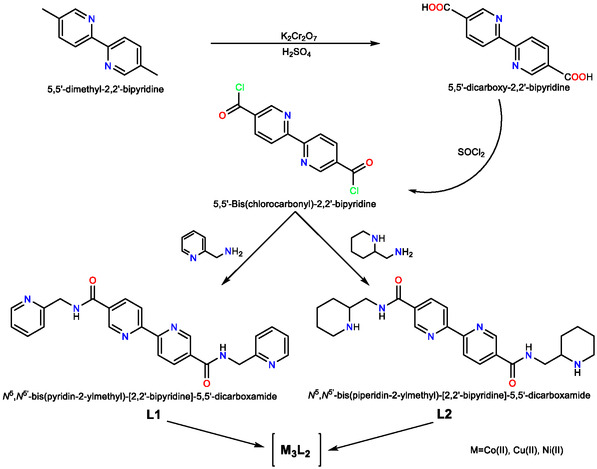
Synthetic routes for the ligands and their corresponding complexes.

The L1 and L2 ligands were synthesized from commercially available 5,5′‐dimethyl‐2,2′‐bipyridine via carboxylation, acylation, and condensation of acyl chloride with the corresponding amine, as shown in Scheme 1 [5]. The metal complexes were obtained by stirring the ligand and metal salts in methanol at a 1:1 ratio at room temperature. The ligands were characterized by FT‐IR (ATR), ^1^H‐NMR, ^13^C‐NMR‐DEPT, ESI‐MS, UV–Vis, and elemental analysis techniques; metal complexes were characterized by FT‐IR, MALDI TOF‐MS, UV–Vis, and Magnetic susceptibility techniques. All analyses of the compounds are listed in the experimental section.

The characteristic C=O stretching frequencies were observed at 1679 cm^−1^ for 5,5′‐dicarboxy‐2,2′‐bipyridine, 1720 cm^−1^ for 5,5′‐bis (chlorocarbonyl)‐2,2′‐bipyridine, 1626 cm^−1^ for L1, 1641 cm^−1^ for L2, and between 1633 and 1657 cm^−1^ for the complexes. The FT‐IR spectra of the metal complexes were systematically compared with those of the free ligands to determine the coordination modes. The characteristic ν(N–H) amide stretching frequencies, observed at 3288 cm^−1^ for L1 and 3313 cm^−1^ for L2, exhibited a significant downfield shift to the 3216–3283 cm^−1^ range across the entire series. This redshift is interpreted as a clear indication of intramolecular N–H…O hydrogen bonding between the amide proton and the carbonyl oxygen. This phenomenon stabilizes the resulting chelate structure and reflects the change in the electronic environment of the amide group during metal coordination [5]. The successful formation of the metal–ligand framework is further supported by the appearance of new vibrational modes in the far‐IR region (200–600 cm^−1^), which are absent in the free ligands. The bands identified in the 524–544 cm^−1^ region across all complexes are assigned to the ν(M–O) stretching vibrations, while the signals appearing in the lower 402–474 cm^−1^ range are attributed to the ν(M–N) stretching modes [2, 9, 23]. Specifically, for Co‐L1 (1), these bands appear at 524 and 414 cm^−1^, confirming the participation of both the amide oxygen and the nitrogen atoms in the metal coordination sphere. These assignments are consistent with established spectroscopic standards for transition‐metal complexes with similar donor sets [24]. In the FT‐IR spectra of all complexes, two characteristic bands corresponding to the asymmetric and symmetric stretching vibrations of the acetate group were observed and for the complexes of the first ligand, *ν*_as_ (COO^−^) and *ν*_s_ (COO^−^) appeared at 1538–1548 and 1384–1410 cm^−1^, respectively, while for the complexes of the second ligand, these bands occurred at 1538–1545 and 1387–1401 cm^−1^. The resulting Δ*ν* values (≈138–159 cm^−1^) are close to those typically reported for ionic acetate, indicating that the acetate groups most likely act as counter anions rather than coordinated ligands [24].

ESI‐MS and elemental analyses of the ligands are consistent with their structures.

NMR spectroscopy was used to determine the proton and carbon resonances of the ligands. In addition, the hydrogen numbers in carbon atoms were determined by a modern NMR technique called DEPT. NMR spectroscopy confirmed the successful synthesis and molecular symmetry of the free ligands. Amide NH protons were observed as triplets at 9.41 ppm in L1. In the L1 ligand, aliphatic CH_2_ protons were observed as a doublet at 4.61 ppm, and the coupling value between aliphatic CH_2_ and amide NH protons was determined as 6 Hz. A critical diagnostic for dicarboxamide formation is the significant downfield shift of the nitrogen‐bound protons; in L1, the amide NH triplet appeared at 9.41 ppm, representing a dramatic shift from the precursor's characteristic 1–3 ppm primary amine range. Internal symmetry was validated by carbon‐13 NMR data, in which L1 and L2 exhibited only 12 unique signals among their 24 total carbons. This symmetry equivalence is a direct result of the C2‐rotational symmetry inherent in the 2,2′‐bipyridine‐5,5′‐dicarboxamide framework, rendering the two halves of the molecule chemically equivalent. Similarly, the ^1^H‐NMR spectrum of L2 displayed signals for 16 of the 32 total protons, consistent with the high degree of symmetry in its bis(piperidin‐2‐ylmethyl) architecture.

**FIGURE 1:**
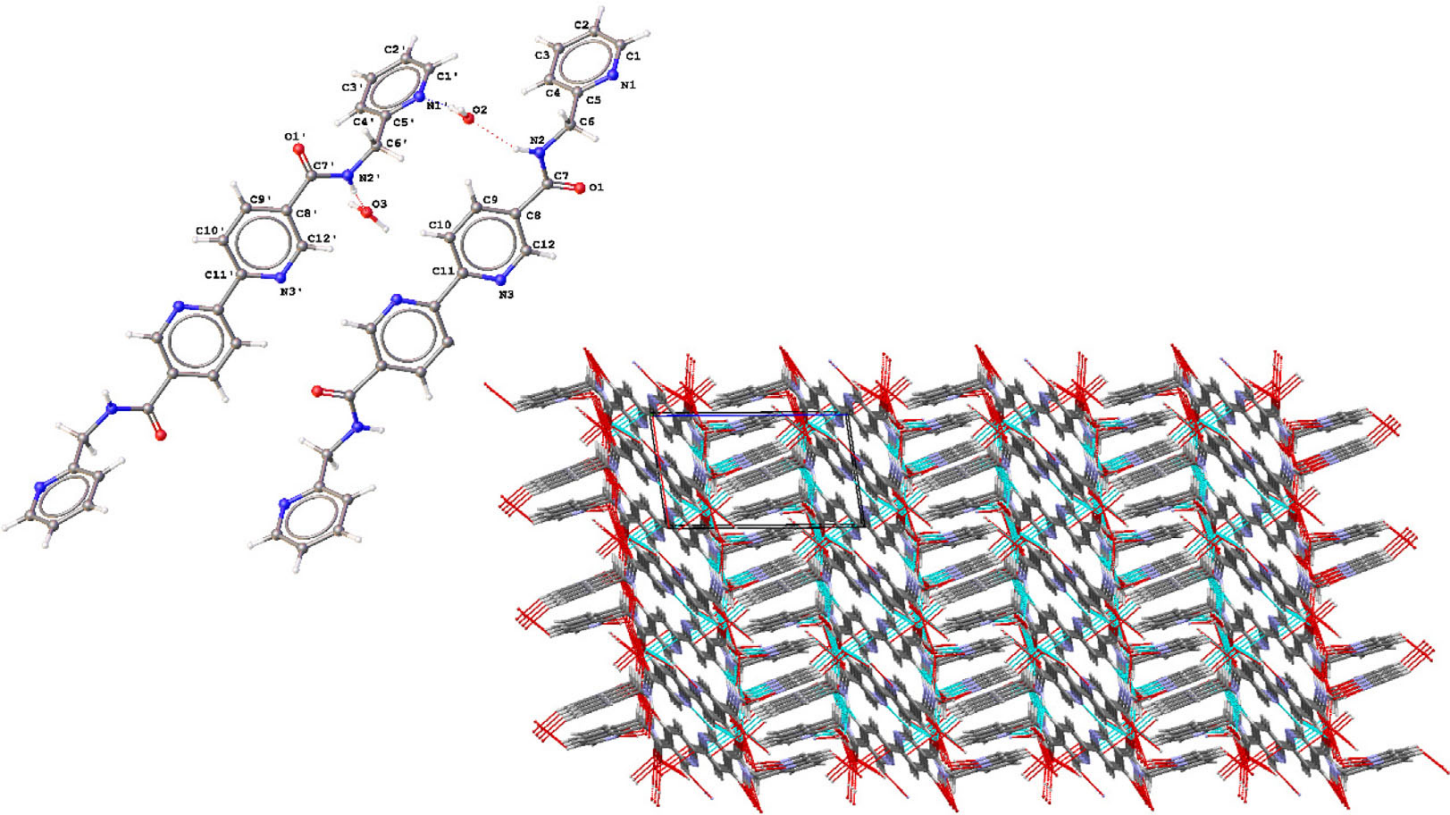
Partially labeled molecular structure and molecular packing view (3 × 3 × 3) for L1.

The details of the crystal data collection and refinement of L1 are summarized in Table 1. The bond distances and angles of the ligand are listed in Table 2. The partially labeled molecular structure and molecular packing view for L1 are shown in Figure 1. L1 crystallized in the space group P‐1 in the triclinic crystal system. The unit cell parameters were determined to be *a* = 7.4577(5) Å, *b* = 11.9258(7) Å, *c* = 13.4931(8) Å, α = 70.639(2)°, β = 79.309(2)°, γ = 81.924(2)°, V = 1108.50(12)Å^3^, Z = 2. In the crystal structure of **L1**, the crystal packing is stabilized by intermolecular hydrogen bonds, CH···O, CH···N, C = O···H, C = O···O, N···O, π··· H, and CO··· π interactions, forming a 3D network (see Figure 1).

**TABLE 2:** Bond lengths and bond angles for L1.

Atom	Atom	Length, Å	Atom	Atom	Length, Å	Atom	Atom	Atom	Angle, °	Atom	Atom	Atom	Angle, °
C1	C2	1.368(3)	C1’	C2’	1.367(3)	N1	C1	C2	124.01(15)	N1’	C1’	C2’	123.74(14)
C1	N1	1.346(2)	C1’	N1’	1.3448(19)	C3	C2	C1	118.04(15)	C3’	C2’	C1’	118.20(15)
C2	C3	1.372(3)	C2’	C3’	1.379(2)	C4	C3	C2	119.27(17)	C4’	C3’	C2’	119.11(16)
C3	C4	1.385(2)	C3’	C4’	1.386(2)	C5	C4	C3	119.17(15)	C5’	C4’	C3’	119.12(15)
C4	C5	1.379(2)	C4’	C5’	1.381(2)	C6	C5	C4	123.11(12)	C6’	C5’	C4’	123.70(12)
C5	C6	1.5098(19)	C5’	C6’	1.509(2)	N1	C5	C4	122.13(13)	N1’	C5’	C4’	122.02(13)
C5	N1	1.3399(17)	C5’	N1’	1.3399(17)	N1	C5	C6	114.68(12)	N1’	C5’	C6’	114.26(12)
C6	N2	1.4546(16)	C6’	N2’	1.4524(17)	N2	C6	C5	115.77(11)	N2’	C6’	C5’	115.35(12)
C7	C8	1.5032(16)	C7’	C8’	1.4986(17)	N2	C7	C8	117.54(11)	N2’	C7’	C8’	116.53(11)
C7	N2	1.3375(16)	C7’	N2’	1.3378(17)	O1	C7	C8	120.73(11)	O1’	C7’	C8’	120.69(12)
C7	O1	1.2351(16)	C7’	O1’	1.2312(16)	O1	C7	N2	121.73(12)	O1’	C7’	N2’	122.77(12)
C8	C9	1.3850(19)	C8’	C9’	1.3901(18)	C9	C8	C7	124.68(11)	C9’	C8’	C7’	119.54(11)
C8	C12	1.3925(18)	C8’	C12’	1.3935(18)	C12	C8	C7	118.02(11)	C12’	C8’	C7’	122.85(11)
C9	C10	1.3823(18)	C9’	C10’	1.3816(18)	C12	C8	C9	117.28(12)	C12’	C8’	C9’	117.56(11)
C10	C11	1.3837(18)	C10’	C11’	1.3920(18)	C10	C9	C8	119.75(12)	C10’	C9’	C8’	119.50(12)
C11	C11^a^	1.491(2)	C11’	C11’^b^	1.491(2)	C11	C10	C9	119.09(13)	C11’	C10’	C9’	118.74(12)
C11	N3	1.3431(17)	C11’	N3’	1.3447(16)	C11^a^	C11	C10	120.58(14)	C11’^b^	C11’	C10’	121.11(14)
C12	N3	1.3409(17)	C12’	N3’	1.3365(16)	N3	C11	C10	122.25(11)	N3’	C11’	C10’	122.65(11)
						N3	C12	C8	123.69(13)	N3’	C12’	C8’	123.80(12)
						C5	N1	C1	117.37(14)	C5’	N1’	C1’	117.81(13)
						C7	N2	C6	121.02(11)	C7’	N2’	C6’	122.03(12)
						C12	N3	C11	117.92(11)	C12’	N3’	C11’	117.63(11)

a 2‐X, 1‐Y, 2‐Z.

b 1‐X, ‐Y, 2‐Z.

The MALDI‐TOF mass spectra of all complexes exhibit a series of peaks corresponding to ligand‐derived ions and various metal–ligand species formed during the desorption/ionization process. In the low‐mass region, signals attributable to protonated and sodiated ligand ions ([L + H]^+^ and [L + Na]^+^) are consistently observed, indicating partial dissociation of the ligand under MALDI conditions. Fragment ions originating from the partial cleavage of the ligand framework are also present in several spectra. In the intermediate‐mass region, the peaks can be assigned to mononuclear metal–ligand species, such as [ML]^+^ and [ML_2_]^+^ (M = Co, Cu, Ni), supporting the formation of metal complexes containing one or two coordinated ligand molecules. In addition to these mononuclear species, several higher m/z signals correspond well with the calculated values for multinuclear aggregates, most plausibly [M_2_L_2_]^+^ and [M_3_L_2_]^+^ type ions. For several of these peaks, the experimentally observed m/z values match the calculated masses when additional small molecules, such as acetate (OAc^−^) and/or water (H_2_O), are included, leading to assignments such as [ML(OAc)]^+^, [ML_2_(H_2_O)]^+^, [M_2_L_2_(OAc)]^+^, [M_2_L_2_(H_2_O)]^+^, [M_3_L_2_(OAc)]^+^, and related solvated or adduct‐containing ions. The detection of these species suggests that, in addition to the principal metal–ligand complexes, partial aggregation of metal centers may occur either in the condensed phase or during the MALDI ionization process. It should be noted that several higher m/z signals observed for the L2‐based complexes appear to correspond to species associated with noninteger solvent molecules, such as 0.5 H_2_O (e.g., for Co‐L2, Cu‐L2, and Ni‐L2). These specific assignments are flagged as tentative MALDI artifacts rather than stable lattice components. Such features are likely the result of complex gas‐phase aggregation or fragmentation processes during high‐energy laser desorption/ionization (MALDI), a phenomenon well documented in the mass spectrometry of multinuclear coordination clusters [5]. Meanwhile, the relatively weak or inconsistent observation of acetate‐containing ions indicates that acetate groups are only weakly associated with the complexes and can be readily lost during ionization, which is consistent with their role as counter anions inferred from the FT‐IR data.

The electronic absorption spectra of the L1 and L2 ligands and their corresponding M_3_L_2_‐type trinuclear Co^+2^, Cu^+2^, and Ni^+2^ complexes were recorded in methanol (10^−5^ M). In all complexes, three distinct absorption bands were observed in the ranges of 202–204 nm, 250–261 nm, and 299–313 nm. These bands can be assigned to intraligand electronic transitions. Specifically, the highest‐energy band is attributed to localized π → π* transitions, possibly overlapping with solvent background attenuation. The mid‐range band is characteristic of aromatic ring π → π* excitations within the aromatic core of the ligands. In contrast, the lowest energy band is likely derived from a combination of n → π* transitions (originating from the carbonyl and heteroatom lone pairs) and possible ligand‐centered charge transfer (CT) processes facilitated by the conjugated pathways. Due to the very high molar absorptivity of these ligand‐dominated excitations, the inherently weak and spin‐forbidden d–d electronic transitions of the metal ions were not observed across the entire 200–800 nm spectral range.

All of the multidentate complexes exhibit high thermal stability, melting only at temperatures above 300 °C, and undergo charring and decomposition above 300 °C.

The room‐temperature magnetic susceptibility measurements were performed for all six complexes using the Gouy method, and the effective magnetic moments (μ_eff_) were calculated at 298 K. The cobalt(II) complexes Co‐L1 and Co‐L2 exhibit μ_eff_ values of 5.08 and 4.98 μB, respectively, consistent with the 4.7–5.2 μB range expected for high‐spin octahedral Co(II) (d^7^, S = 3/2). The observed enhancement above the spin‐only value (3.87 μB) is attributable to the significant orbital contribution associated with the ^4^T_1_g ground term. The nickel(II) complexes Ni‐L1 and Ni‐L2 display μ_eff_ values of 3.06 and 2.97 μB, slightly above the spin‐only value of 2.83 μB and within the range of 2.9–3.4 μB anticipated for high‐spin octahedral Ni(II) (d^8^, S = 1, ^3^A_2_g ground term). The copper(II) complexes Cu‐L1 and Cu‐L2 show μ_eff_ values of 2.06 and 2.01 μB, in good agreement with the 1.7–2.2 μB range expected for Cu(II) (d^9^, S = 1/2) in a Jahn–Teller‐distorted octahedral environment, where a modest spin–orbit contribution is commonly observed. Across all three metal series, the close similarity of μ_eff_ values between the L1 and L2 complexes indicates that the two ligands impose comparable coordination environments. The magnetic data are fully consistent with the proposed M_3_L_2_ stoichiometry featuring three high‐spin divalent metal centers in octahedral geometries, corroborating the MALDI‐TOF mass spectrometric results.

Consequently, the comprehensive characterization data strongly support the formation of the proposed trinuclear complexes with an overall [M_3_L_2_(H_2_O)_*n*_]⋅6OAc stoichiometry.

### 3.2 Antimicrobial Activity Data

#### 3.2.1 Antimicrobial Activity Data by Macrodilution

The results for L1, L2, Co‐L1, Cu‐L1, Ni‐L1, Co‐L2, Cu‐L2, Ni‐L2, L1, and L2 are presented in Table 3. Agar plates were inspected after 24 h of incubation to assess bacterial growth. In dishes where no bacterial growth was observed, the substance concentration was considered sufficient to inhibit bacterial growth.

**TABLE 3:** Antimicrobial activity was determined by the macrodilution method.

	*S. aureus*	*E. coli*	*P. aeruginosa*
**Gentamicin (Ref.)**	−	−	−
**Positive control**	+	+	+
**L1**	+	+	+
**Co‐L1**	+	+	+
**Cu‐L1**	+	+	+
**Ni‐L1**	+	+	+
**L2**	+	+	+
**Co‐L2**	−	−	−
**Cu‐L2**	+	+	+
**Ni‐L2**	+	+	+

#### 3.2.2 Antimicrobial Activity Data by MIC

Bacterial growth was quantified by measuring the absorbance at 600 nm using a spectrophotometer. Data were analyzed using Microsoft Excel to calculate MIC values. All assays included positive controls (McFarland 0.5 adjusted culture) and negative controls (DMSO), as shown in Table 4. Table 4 presents the spectrophotometric measurements obtained at various time points for bacterial species exposed to Co‐L2, along with positive and negative controls.

**TABLE 4:** Spectrophotometric measurements at different time points for the bacterial strains containing Co‐L2.

	0 h	2 h	4 h	6 h	24 h	30 h	48 h
Negative control (NC)	0.067	0.066	0.065	0.064	0.064	0.064	0.064
Positive control (PC) *E. coli*	0.072	0.072	0.074	0.082	0.259	0.289	0.407
**Co‐L2** (500 ppm), *E. coli*	0.059	0.061	0.063	0.064	0.066	0.068	0.067
**Co‐L2** (1000 ppm), *E. coli*	0.062	0.065	0.067	0.069	0.070	0.070	0.071
**Co‐L2** (2000 ppm), *E. coli*	0.077	0.080	0.082	0.083	0.090	0.089	0.090
PC *P. aeruginosa*	0.061	0.065	0.075	0.093	0.384	0.439	0.647
**Co‐L2** (500 ppm), *P. aeruginosa*	0.065	0.064	0.067	0.068	0.074	0.076	0.076
**Co‐L2** (1000 ppm), *P. aeruginosa*	0.065	0.065	0.066	0.067	0.070	0.070	0.070
**Co‐L2** (2000 ppm), *P. aeruginosa*	0.077	0.079	0.081	0.083	0.087	0.087	0.087
PC *S. aureus*	0.063	0.064	0.067	0.075	0.267	0.300	0.411
**Co‐L2** (500 ppm) *S. aureus*	0.057	0.058	0.059	0.060	0.060	0.061	0.062
**Co‐L2** (1000 ppm) *S. aureus*	0.062	0.063	0.065	0.065	0.067	0.068	0.068
**Co‐L2** (2000 ppm) *S. aureus*	0.074	0.076	0.078	0.077	0.080	0.081	0.082

The quantitative evaluation showed that Co‐L2 suppressed bacterial growth with an MIC of 500 μg/mL against all tested strains. However, this value is higher than that of the standard antibiotic Gentamicin (which exhibited MIC values of 4–8 μg/mL for these ATCC strains) [21]. The activity of Co‐L2 is significant, given that it is a novel coordination complex targeting resistant phenotypes. The consistent MIC value of 500 μg/mL across both Gram‐positive and Gram‐negative species highlights the broad‐spectrum potential of the Co‐L2 complex.

The initial results for all three bacterial species indicated that most compounds had no inhibitory effect, as bacterial growth was similar to that in the positive control. Based on the growth patterns in Table 3, *E. coli*, *P. aeruginosa*, and *S. aureus* continued to grow in the presence of Co‐L1, Cu‐L1, Ni‐L1, Cu‐L2, and Ni‐L2 at various concentrations, indicating that these compounds did not inhibit bacterial growth.

In contrast, Co‐L2 exhibited significant antimicrobial activity against all three bacterial species. As shown in Tables 3 and 4, even at the lowest concentration tested (500 ppm), Co‐L2 effectively reduced bacterial growth in *E. coli*, *P. aeruginosa*, and *S. aureus*.

Based on the spectrophotometric growth data presented in Table 4, the MIC for the Co‐L2 complex was determined to be 500 ppm against all tested strains (*E. coli*, *P. aeruginosa*, and *S. aureus*). At this concentration, no significant increase in OD_600_ was observed over the 48‐h incubation period, with values remaining consistent with the negative control. In contrast, for all other complexes, the MIC values were >2000, as they failed to inhibit bacterial growth at the highest concentration tested.

The significant antimicrobial activity of Co‐L2 against all tested species (Table 4) can be attributed to its enhanced lipophilic nature. According to Tweedy's chelation theory and the Overtone concept, metal coordination reduces the polarity of the metal ion by delocalizing charge, thereby increasing the lipophilicity of the complex [25]. This lipophilic character facilitates the complex's penetration through the lipid bilayer of the bacterial cell membrane. Recent studies on bipyridine‐based metal complexes have experimentally demonstrated that such compounds compromise cytoplasmic membrane integrity, leading to increased cell permeability [4]. Furthermore, visual evidence from SEM analysis confirms that related coordination frameworks can physically rupture the bacterial membrane, leading to cell lysis [3]. However, the exact mechanisms by which Co‐L2 may affect human health, given its physical and chemical properties, remain unclear and require further investigation.

#### 3.2.3 Inhibition Results

**FIGURE 2:**
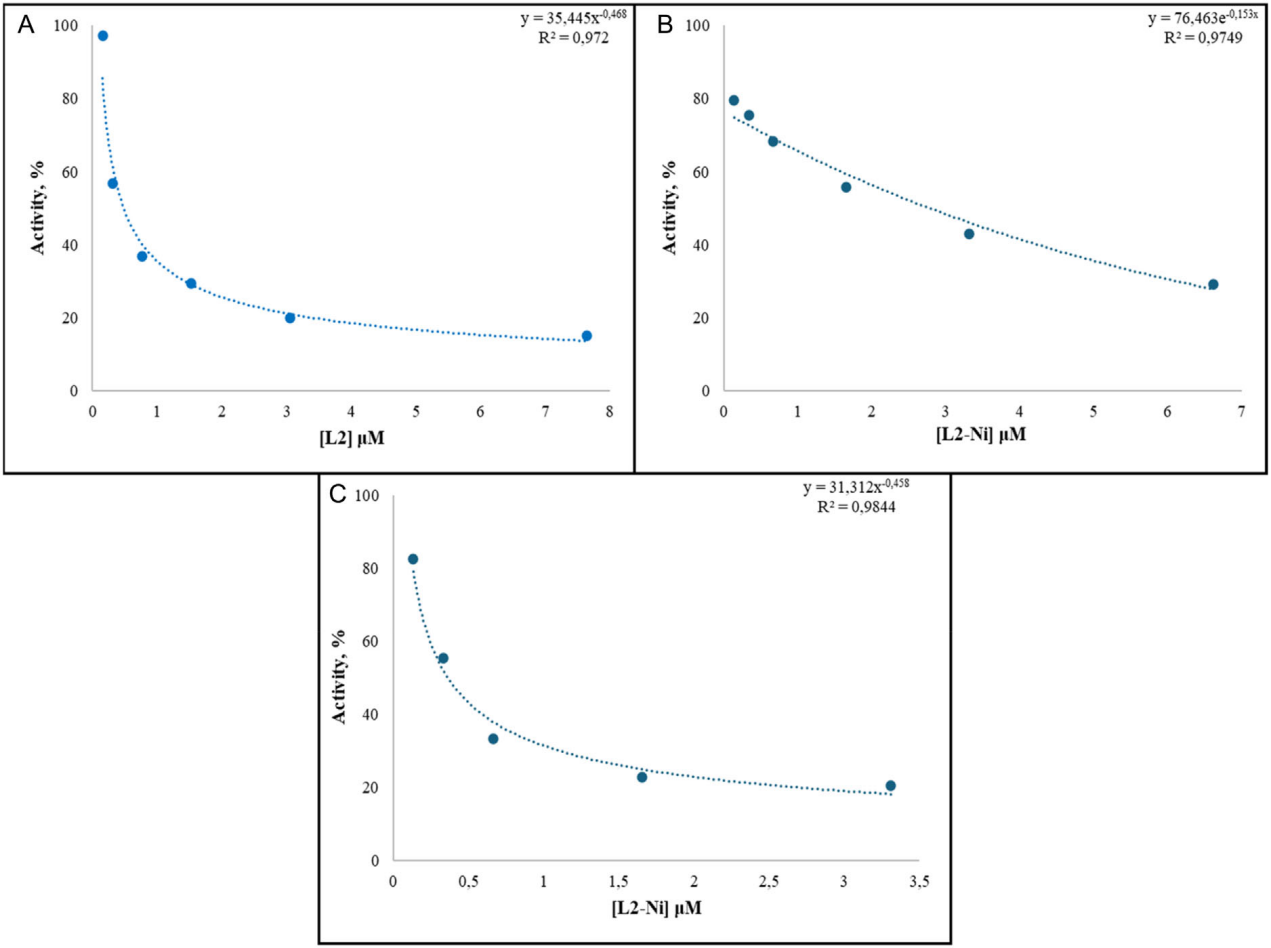
Activity (%)‐concentration graphs for molecules showing the strongest inhibitory effect. (A) Activity (%) versus concentration graph for AChE inhibition by the L2 ligand; (B) activity (%) versus concentration graph for BChE inhibition by the L2‐Ni complex; and (C) activity (%) versus concentration graph for pancreatic lipase inhibition by the L2‐Ni complex.

The data obtained from studies aimed at determining the effect of ligands and complexes on enzyme activities are summarized in Table 5. The results showed that the L2 ligand exhibited a stronger inhibitory effect than the other substances on AChE and Ni‐L2 for pancreatic lipase and BChE (Figure 2). However, the L1 ligand did not affect lipase, and the Co‐L1 complex had no effect on any of the enzymes studied. Compared with the previously reported effect of N^4^, N^4^’‐bis(piperidin‐2‐ylmethyl)‐[2,2′‐bipyridine]‐4,4′‐dicarboxamide ligand [5], this study determined that the L2 ligand exhibited a stronger inhibitory effect, particularly on AChE and BChE enzymes. In another study, the 2,2′‐bipyridine‐Ni complex was found to inhibit AChE with an IC50 of 14 μM [26]. In this study, the Ni‐L2 complex showed a stronger inhibitory effect on AChE, with an IC50 of 1.03 μM. In a study that determined the inhibitory effects of Schiff base ligands containing a pyridine group and the metal complexes synthesized with these ligands on pancreatic lipase, the IC50 value of orlistat, an effective lipase inhibitor used in the treatment of obesity, was reported as 58.4 ± 3.2 μM [9] and compared with this value, the ligands and complexes used in this study, which showed inhibitory effects on lipase, appeared more effective. In this respect, the Ni‐L2 complex stood out. One noteworthy point is that ligands sometimes exhibit a stronger inhibitory effect on enzymes than the metal complex. This phenomenon may be explained by the fact that ligands may be more biologically active in complexes containing a metal center that does not directly bind to the enzyme [27].

**TABLE 5:** For ligands and complexes, studied concentration ranges and IC50 values.

	Concentration range, μM			**IC**_**50**_ **values, μM**		
	Lipase	AChE	BChE	Lipase	AChE	BChE
**L1**	1.57–314.27	1.57–314.27	3.77–377.12	—	144.2	147.2
**L2**	1.83–91.63	0.15–7.64	1.83–36.65	24.08	0.479	5.04
**Cu‐L1**	0.18–22.16	0.18–44.33	0.18–44.33	9.50	9.63	21.00
**Co‐L1**	0.13–15.65	0.31–6.26	0.13–15.65	—	—	—
**Ni‐L1**	0.13–33.09	0.09–11.03	0.33–16.55	15.19	5.373	24.76
**Cu‐L2**	0.17–8.63	0.17–8.63	0.17–21.57	3.21	2.15	5.42
**Co‐L2**	0.14–17.48	0.14–6.99	0.35–17.48	9.78	1.43	10.05
**Ni‐L2**	0.13–3.31	0.09–2.21	0.13–6.62	0.36	1.03	4.53

#### 3.2.4 Docking Results

**FIGURE 3:**
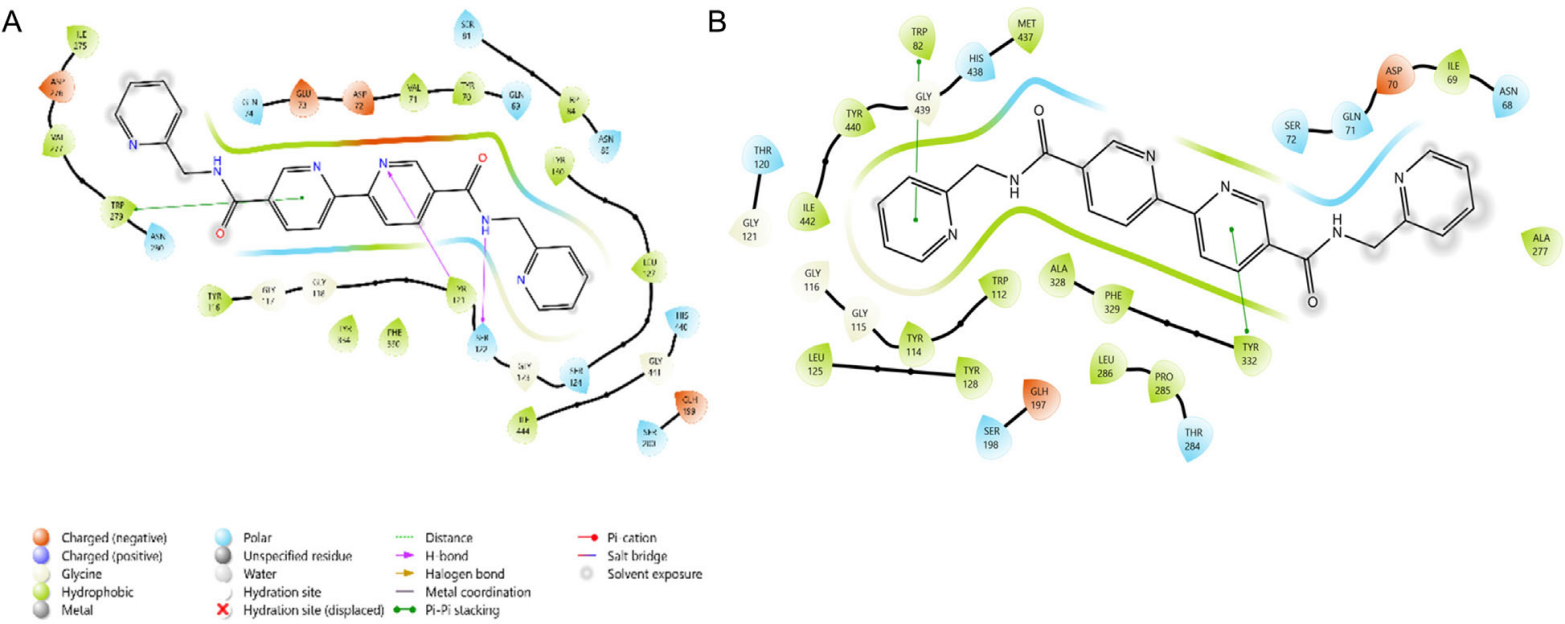
2D images of the interactions between AChE (A) and BChE (B) enzymes and L1.

The enzyme–inhibitor interaction mechanism for molecules exhibiting inhibitory effects was elucidated through docking studies. However, because the complexes were large, the ligand preparation process could not be performed using the docking program; therefore, the studies were conducted only with the ligands. The results showed that there were hydrophobic interactions between L1 and the AChE residues TYR70–116–121–130–334, VAL71–277, TRP84–279, LEU127, ILE275–444, and PHE330. Negatively charged interactions occurred between the ligand and the residues ASP72–276, GLU73, and GLH199. Polar interactions occurred with residues GLN69–74, SER81–122–124–200, ASN85–280, and HIS440. Hydrogen bonding occurred between the ligand and the TYR121 and SER122 residues of the AChE enzyme, while a π–π stacking interaction occurred with the TRP279 residue. The binding score for the ligand–enzyme interaction was −6.986 (Table 6 and Figure 3A).

**TABLE 6:** Binding scores (kcal/mol) for ligands and standard enzyme inhibitors.

		Docking score	XP GScore	Glide emodel
**AChE**	L1	−6.986	−7.002	−86.779
	L2	−11.053	−11.053	−82.296
	Tacrine	−9.670	−9.671	−46.072
**BChE**	L1	−4.841	−4.857	−74.611
	L2	−4.825	−4.825	−63.002
	Tacrine	−6.913	−6.913	−42.707
**Lipase**	L1	—	—	—
	L2	−2.774	−2.775	−39.718
	Methoxy phosphinic acid	−0.863	−0.863	−20.917

Hydrophobic interactions occurred between L1 and BChE residues Ile69–442, Trp82–112, Tyr114–128–332–440, Leu125–286, Ala277–328, Pro285, Phe329, and Met437. Negatively charged interactions occurred with Asp70 and Glu197. Polar interactions occurred with Asn68, Glu71, Ser72–198, Thr120–284, and His438. π–π stacking interactions occurred between the enzyme residues Trp82 and Tyr332 and the ligand. The docking score for the ligand–enzyme interaction was −4.841, which was lower than that for tacrine (−6.913) (Figure 3B).

**FIGURE 4:**
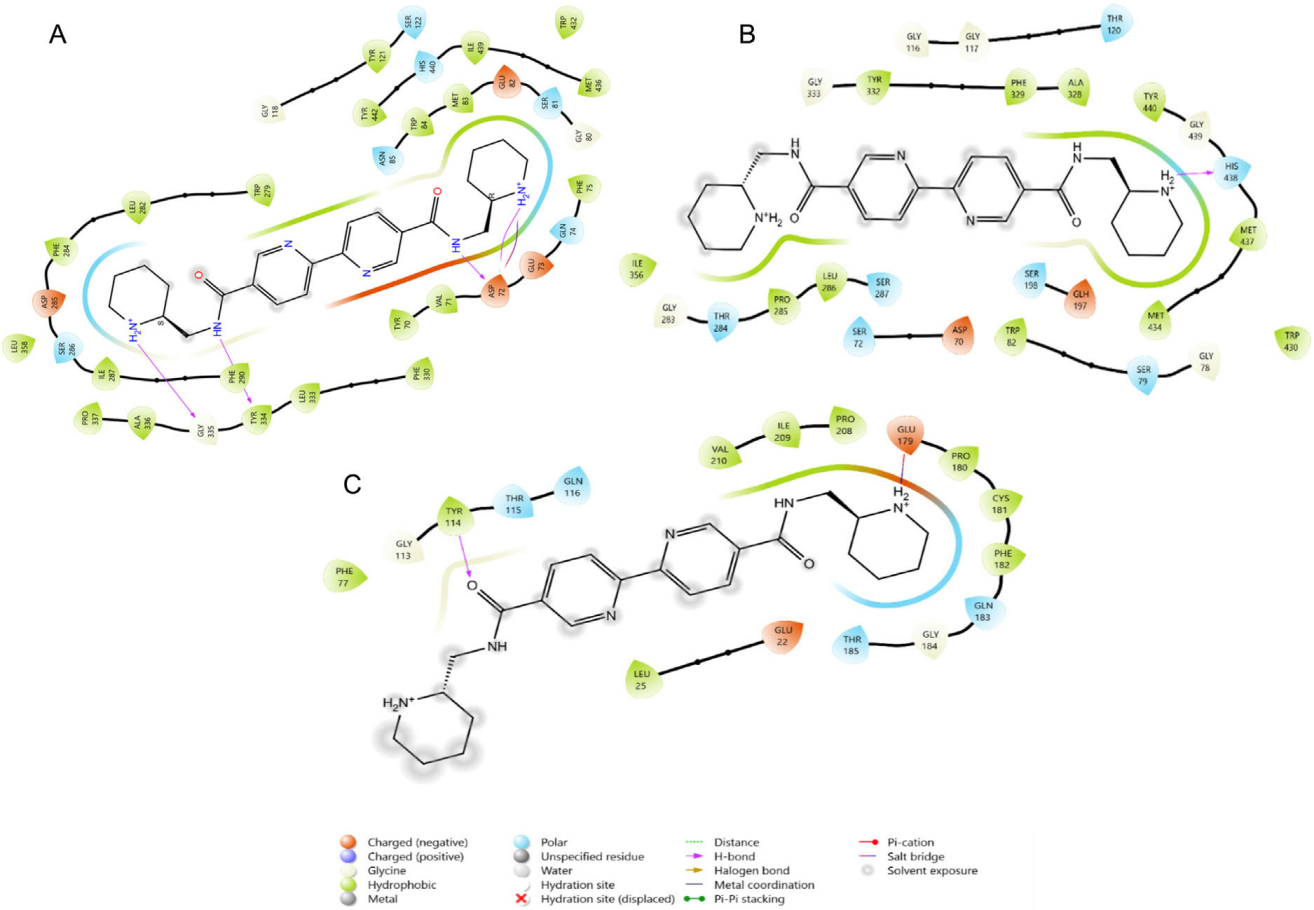
2D images of the interactions of acetylcholinesterase (A), butyrylcholinesterase (B), and pancreatic lipase (C) with L2.

Hydrophobic interaction occurred between the L2 and AChE residues TYR70 −121–334–442, VAL71, PHE75–284–290–330, MET83–436, TRP84–279–432, LEU282–333–358, ILE287–439, ALA336, and PRO337. Negatively charged interactions occurred with residues ASP72−285 GLU73−82, and ASP285, and polar interactions occurred with GLN74, SER81–122−286, ASN85, and HIS440. An H‐bond occurred between ASP72 and TYR334, and a salt bridge formed between ASP72. The ligand–enzyme interaction binding score was −11.053 (Figure 4A). Compared with that for tacrine (−9.670), the interaction appears stronger. The catalytic region of the enzyme consists of Ser200, His440, and Glu327 located in the esteratic subregion, with Ser200 responsible for initiating the catalytic process [28]. Polar interactions between the L1 ligand and Ser200 and His440, and between the L2 ligand and His440, may be effective in inhibition. Hydrophobic interactions ensued between L2 and BChE residues TRP82−430, PRO285, LEU286, TYR332‐440, PHE329, ALA328, ILE356, and MET434−437. Negatively charged interactions occurred with residues ASP70 and GLH197; polar interactions ensued with residues SER72–79–198−287, THR120−284, and HIS438. In addition, an H‐bond interaction occurred with HIS438. The docking score for the ligand–enzyme interaction was −4.825, lower than that of tacrine (−6.913) (Figure 4B). Hydrophobic interactions were observed between L2 and specific residues of pancreatic lipase, including LEU25, PHE77‐182, TYR114, PRO180‐208, CYS181, ILE209, and VAL210. A negatively charged interaction was detected with residues GLU22 and 179, and a polar interaction was observed with residues THR115185 and GLN116–183. A salt bridge occurred with GLU179. The docking score for the ligand–enzyme interaction was −2.774, surpassing that of the standard inhibitor, methoxy phosphinic acid, which had a score of −0.863 (Figure 4C).

## 4 Conclusion

In this study, two novel 2,2′‐bipyridine‐5,5′‐dicarboxamide ligands, N5, N5′‐bis (pyridin‐2‐ylmethyl)‐[2, 2′‐bipyridine]‐5, 5′‐dicarboxamide (L1) and N5, N5′‐bis (piperidin‐2‐ylmethyl)‐[2,2′‐bipyridine]‐5, 5′‐dicarboxamide (L2), were synthesized and characterized. Complexes of these ligands with Co(II), Cu(II), and Ni(II) were also prepared. The structure of L1 was determined using single‐crystal X‐ray diffraction. The antibacterial properties of the ligands and their metal complexes were evaluated against resistant bacterial strains. Quantitative antibacterial evaluation revealed that the Co‐L2 complex possesses significant potency, with a determined MIC value of 500 against both Gram‐positive and Gram‐negative resistant strains. This was confirmed by time‐dependent absorbance measurements, which showed that Co‐L2 completely suppressed bacterial proliferation at the lowest tested dosage. Future studies should focus on understanding its mechanism of action and evaluating its in vivo efficacy to assess its therapeutic potential fully. The complexes Co‐L1, Cu‐L1, Ni‐L1, Cu‐L2, and Ni‐L2 did not show effectiveness against the tested bacteria. Testing at higher concentrations is recommended to identify effective options.

Additionally, enzyme inhibition studies were performed, highlighting the potential therapeutic applications of these complexes in enzyme‐targeted therapies. Among the complexes, Ni‐L2 demonstrated the most significant inhibitory effect against pancreatic lipase and BChE. However, the free L2 ligand exhibited the highest potency toward AChE. These results emphasize that 5,5′‐regiochemistry and the type of pendant arms are decisive factors in biological efficacy. The current study demonstrates that the 5,5′‐regioisomers exhibit biological potencies comparable and consistent with those of the 4,4′‐analogs, confirming that the high broad‐spectrum antimicrobial efficacy and enzyme inhibitory profile are robustly maintained across different regiochemical frameworks.

## Funding

This study was supported by Karabük Üniversitesi (FYL‐2020‐2264).

## Conflicts of Interest

The authors declare no conflicts of interest.

## Data Availability

The data that support the findings of this study are available from the corresponding author upon reasonable request.
